# Genomics of Major Crops and Model Plant Species

**DOI:** 10.1155/2008/171928

**Published:** 2009-03-16

**Authors:** P. K. Gupta, Yunbi Xu

**Affiliations:** ^1^Molecular Biology Laboratory, Department of Genetics and Plant Breeding, Chaudhary Charan Singh University, Meerut, 250 004, India; ^2^International Maize and Wheat Improvement Center (CIMMYT), Apartado Postal 6-64, 06600 Mexico, DF, Mexico

Plant genomics research had its beginning in December 2000, with the publication of the whole genome sequence of the model plant species *Arabidopsis thaliana*. Rapid progress has since been made in this area. The significant developments include the publication of a high-quality rice genome sequence in August 2005, draft genome of poplar in September 2006, whole genome sequence of two grapevine genotypes in 2007, and that of transgenic papaya in 2008. Draft sequences of corn gene-space and those of the genomes of *Lotus japonicus* and *Glycine max* have also become available in 2008. Genomes of several other plant species (e.g., *Sorghum bicolor, Manihot esculenta* (cassava), barley, wheat, potato, cotton, tomato, maize, *Brachypodium distachyon* (a small model grass genome), *Medicago truncatula*, shepherd's purse, peach) are also currently being sequenced. Multinational genome projects on *Brassica* and Solanaceous genomes are also in progress. In still other cases (e.g., wheat, corn, barley), where the large genome size prohibits whole genome sequencing, the gene rich regions (GRRs) of the genomes are being identified to bring down the sequencing work to a manageable level. The 10-year-old US National Plant Genome Initiative (NPGI) also made a call for more plant genomes to be sequenced. While making a choice for additional plant genomes to be sequenced, it has also been emphasized that much of plant diversity is available in tropical plants so that during the next decade, more genomes from tropics (e.g., *Carica, Saccharum, Psychoria, Opuntia*) need to be sequenced.

The sequencing information obtained as above will be utilized for both basic and applied research so that while this will help in elucidating evolutionary relationships and developing better phylogenetic classification, this will also help in the discovery of new genes, allele-mining, and large-scale SNP genotyping. In order to achieve these objectives, there has also been a call for sequencing genomes of diverse cultivars of each crop like rice. As a result, the concept of plant pan genome (initially developed for microbial genomes), each composed of “core genome” and “dispensable genome,” has also been introduced. The sequence information from diverse cultivars in a crop will be utilized for molecular breeding. For instance, new technologies have been used for the improvement of *indica* rice, but similar efforts are now being made for improvement of *japonica* rice also. An overview of the present status of plant genomics research and its impact is also available in a recent special issue of Science (April 25, 2008).

The future plant genomics research will certainly derive benefit from the recent development of new-generation sequencing technologies. These new technologies include improvements in sequencing systems based on Sanger's sequencing approach, as well as a number of non-Sanger sequencing technologies that became available during 2005–2008. The non-Sanger technologies include both sequencing based on amplified DNA molecules, and those based on single DNA molecules including Helicos true single molecule sequencing (tSMS) technology commercially launched in 2008. These new-generation sequencing technologies will certainly help in plant genomics research in a big way and may include a variety of research projects. While more plant genomes will be sequenced, epigenomes, transcriptomes, and metabolomes will also be worked out with much higher speed and at a cost reduced by several orders in magnitude. The science of plant genomics will also be influenced by the new emerging areas of “chemogenomics” and “synthetic genomics.”

This special issue of the International Journal of Plant Genomics is devoted to “Genomics of Major Crops and Model Plant Species” with the aim to present an updated account of the genomics of major crop species and the model plant species. Articles published in this special issue involve almost all fields of genomics, including structural genomics, functional genomics, proteomics, metabolomics, and comparative genomics. Discussions also extend to cover phenomics, bioinformatics, epigenetics, and organellar genomics. Translational genomics from model plant species to cultivated crops and applications of genomics in crop improvement are topics for several articles. Structural genomics, as a major field for most crop plants, received a greater attention in this special issue, compared to other fields, including various types of molecular markers from RFLP to SNP and their use in construction of genetic, cytogenetic, and physical maps, QTL/gene mapping, genome sequencing, and generation of genomics resources. Functional genomics is the second field that received more attention, and some issues addressed significantly include gene isolation through map-based cloning and candidate gene approach, as well as functional analysis through insertional mutagenesis, RNAi, TILLING, and transcription profiling.

There are 14 review articles in this special issue, seven belonging to grass family, two devoted to legumes (soybean and *Medicago*), one devoted to oil-seed crop (*Brassica rapa*), and one each to cotton, tomato, potato, and *Citrus*. The special issue starts with several articles on genomics of food crops including wheat, barley and rice. There is a comprehensive article on wheat genomics written by P. K. Gupta et al. (Meerut, India) followed by an article giving an overview on barley genomics by N. Sreenivasulu et al. from IPK (Gatersleben, Germany). On rice genomics, there are two articles: one with emphasis on genome sequencing (written by T. Matsumoto et al. (Japan)) gives an account of international collaboration in sequencing rice genome and its annotation (including structure and composition of rice centromeres and telomeres), and the other on rice molecular breeding (written jointly by B. Collard (Queensland, Australia) and the rice genomics group (including D. J. Mackill) from International Rice Research Institute (IRRI) (Manila, Philippines)) gives a detailed account of how rice genomics resources can be utilized for molecular breeding. A. H. Paterson has written a review on *Sorghum* genomics (giving information on both markers and whole genome sequencing) and H. Budak et al. (from Turkey and Spain) give an updated account of the development of genomics resources for the grass genus *Brachypodium*, which is being preferred over the rice genus *Oryza* as a model for temperate grasses (including cereals and forage grasses). G. M. Souza et al. (Brazil) have written an article on sugarcane functional genomics, outlining the development and the use of ESTs and cDNA microarrays for gene discovery.

Among legume species, soybean (*Glycine max*) is an important crop world-wide, while *Medicago truncatula* and *Lotus japonicus* emerged as model systems for legume biology during the last decade. Therefore, one article on soybean genomics and another on *Medicago truncatula* have been included in this special issue. D. A. Lightfoot from The Illinois Soybean Center (Illinois, USA) discusses the use of Forresr cultivar for the development of genomic resources in this crop. Similarly, Julia Frugoli (SC, USA) with his two other colleagues elsewhere wrote an article on *Medicago truncatula* giving an updated account on the developments of genomic resources in this model legume. C. P. Hong et al. report the current understanding of the genome structure of *Brassica rapa* and efforts for the whole-genome sequencing of the species.

Hong-Bin Zhang from College Station (Texas, USA) and his coworkers (from University of Georgia and China) discuss advances on genomics research in cotton, highlighting the development of DNA marker linkage/physical maps, QTL mapping, ESTs, and whole genome sequencing. The last three articles deal with genomics of two related Solanaceous crops, namely, tomato and potato, as well as a fruit-tree genus (*Citrus*). L. Frusciante et al. from (Portici and Roma, Italy) give an updated account of tomato genomics, G. J. Bryan and I. Hein from Scottish Crop Research Institute (SCRI, Dundee, UK) give an account of potato genomics, and M. Talon (Valencia, Spain) with F. G. Gmitter (Citrus Research and Education Center, University of Florida, USA) give an account for *Citrus* genomics.

